# Fast multiple sequence alignment via multi-armed bandits

**DOI:** 10.1093/bioinformatics/btae225

**Published:** 2024-06-28

**Authors:** Kayvon Mazooji, Ilan Shomorony

**Affiliations:** Department of Electrical and Computer Engineering, University of Illinois Urbana-Champaign, Urbana, IL 61801, United States; Department of Electrical and Computer Engineering, University of Illinois Urbana-Champaign, Urbana, IL 61801, United States

## Abstract

**Summary:**

Multiple sequence alignment is an important problem in computational biology with applications that include phylogeny and the detection of remote homology between protein sequences. UPP is a popular software package that constructs accurate multiple sequence alignments for large datasets based on ensembles of hidden Markov models (HMMs). A computational bottleneck for this method is a sequence-to-HMM assignment step, which relies on the precise computation of probability scores on the HMMs. In this work, we show that we can speed up this assignment step significantly by replacing these HMM probability scores with alternative scores that can be efficiently estimated. Our proposed approach utilizes a multi-armed bandit algorithm to adaptively and efficiently compute estimates of these scores. This allows us to achieve similar alignment accuracy as UPP with a significant reduction in computation time, particularly for datasets with long sequences.

**Availability and implementation:**

The code used to produce the results in this paper is available on GitHub at: https://github.com/ilanshom/adaptiveMSA.

## 1 Introduction

Multiple sequence alignment (MSA) is a central problem in computational biology with applications that include phylogeny inference (Morrison and Ellis 1997), detection of remote homology between protein sequences, protein structure and function inference (Bork and Koonin 1998, Ju *et al.* 2021), and DNA data storage (Antkowiak *et al.* 2020). While significant progress in MSA algorithms has been made in recent years, achieving high alignment accuracy on very large datasets in a computationally efficient manner remains a challenge.

One algorithm that has been shown to produce high-quality alignments on large datasets is ultra-large alignments using phylogeny-aware profiles (UPP) (Nguyen *et al.* 2015). In particular, UPP has been shown to produce higher-quality alignments than other algorithms on large datasets with high levels of sequence length heterogeneity, while giving similar levels of performance on large datasets with little sequence length heterogeneity. While UPP gives improved alignment accuracy on large datasets, it is often slower than other widely used software packages such as MUSCLE (Edgar 2004), MAFFT (Katoh and Toh 2007), and Clustal-Omega (Sievers *et al.* 2011).

**Figure 1.**
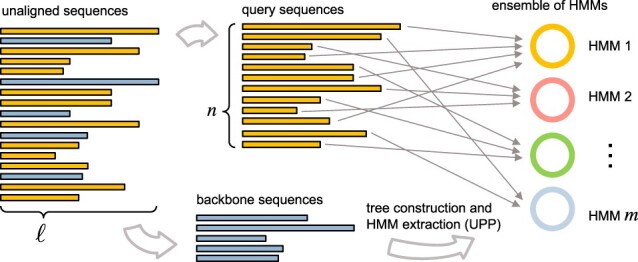
High-level description of the UPP pipeline. The input sequences are split into two parts, the backbone sequences and the query sequences. An alignment and tree are estimated for the backbone sequences, and an ensemble of HMMs is constructed based on the backbone alignment and tree. This is followed by a query-to-HMM assignment step, which in principle requires computing the probability that each HMM could have generated each query sequence.

At a high level, UPP begins by creating an initial alignment and a maximum likelihood (ML) tree from a subset of the input sequences called backbone sequences. These backbone sequences are selected randomly from the set of input sequences that are close to the median input sequence length. All sequences that are not part of the backbone are called query sequences. The ML tree is then decomposed to form sets of related sequences. For each of these sets of sequences, a hidden Markov model (HMM) is formed from its multiple alignment using HMMer (Finn *et al.* 2011). This yields an ensemble of HMMs, as illustrated in Fig. 1. Next, each query sequence is assigned to the HMM that has the highest probability of generating it. For each HMM, the assigned query sequences are added to the alignment corresponding to the HMM using HMMer, one by one. The resulting alignment for each HMM is then merged with the backbone alignment, producing an MSA for the full set of sequences.

For large datasets, the query-to-HMM assignment step is by far the most time-consuming task in UPP. This is because, for each query sequence and each HMM, the probability of the HMM producing the sequence is calculated in $O(\ell^{2})$ time where ℓ is the (maximum) input sequence length. If there are *n* query sequences and *m* HMMs, the query-to-HMM assignment step takes $O(nm\ell^{2})$ time. In a recent work (Park *et al.* 2023), a new algorithm named UPP2 (Park *et al.* 2023) was designed to speed up the query-to-HMM assignment step. For each query sequence, UPP2 only computes the probability for certain HMMs, chosen according to the structure of the ML tree. This reduces the run-time to $O(n\;\text{log}(m)\ell^{2})$, which leads to very substantial time savings, at the price of a small decrease in alignment quality.

In this work [originally developed independently and without knowledge of Park *et al.* (2023)], we pursue a different route to speed up the query-to-HMM assignment step. Rather than reducing how many HMMs each query sequence is compared against, we reduce how much computation is spent in each query-to-HMM comparison. To do so, we introduce two algorithmic ideas:

1. A new *k*-mer-based similarity score J(q,h) that works as a proxy for the probability that a query sequence *q* was generated by HMM *h*. We refer to J(q,h) as the *J*-score. Notably, J(q,h) can be efficiently estimated by sampling *k*-mers in time sublinear in ℓ.
2. We leverage the fact that J(q,h) can be estimated using random *k*-mer samples to propose an adaptive estimation framework for finding $\arg\;\max_{h}J(q,h)$. We take inspiration from the recent literature on using Multi-Armed Bandits (MABs) to speed up large-scale computations via adaptivity (Bagaria *et al.* 2018, 2021, Heckel *et al.* 2018, Tiwari *et al.* 2020, Kamath *et al.* 2020).

**Figure 2.**
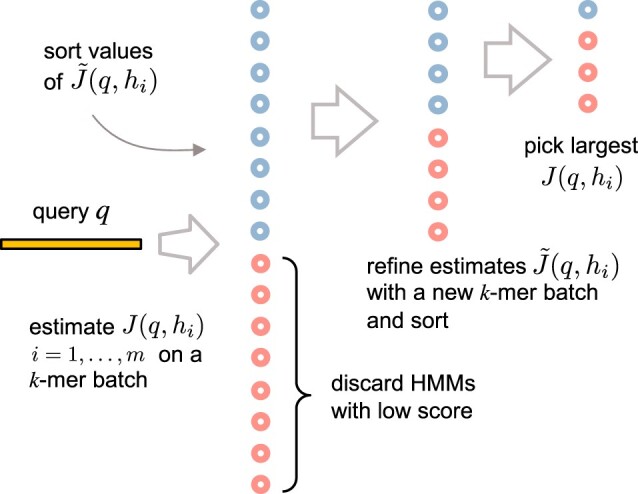
Adaptive search for the HMM $h_{i}$, i=1,…,m, that maximizes $J(q,h_{i})$. We first estimate the similarity score J(q,h) for each HMM based on a random *k*-mer batch, and discard HMMs with a low score. The score for the remaining HMMs is refined based on a new *k*-mer batch, and this process can be repeated. In the end, the exact value of J(q,h) is computed for a small number of HMMs, and the best one is chosen.

An overview of the adaptive search for $\arg\;\max_{h}J(q,h)$ is shown in Fig. 2. By drawing random subsets of *k*-mers, estimates of the score J(q,h) can be efficiently computed. This allows for iterative refinement of estimates of J(q,h) for more promising HMMs. Building on theoretical results for MABs, we show that using the Upper Confidence Bound algorithm (Lai and Robbins 1985), it is possible to identify $\arg\;\max_{h}J(q,h)$ with high probability in time O(mn log m). However, for our practical implementation, we opt for an algorithm based on the Sequential Halving MAB algorithm (Karnin *et al.* 2013). This implementation runs in time O(mn + mℓ) and achieves very good performance. In particular, when used in the UPP pipeline, it reduces the overall run-time substantially for datasets containing long sequences (with similar alignment accuracy), even when compared to UPP2.

## 2 Adaptive search for best HMM

As described in Section 1, our approach for accelerating UPP is based on a new similarity metric, the *J*-score, which admits an adaptive search for $\arg\;\max_{h}J(q,h)$. In Sections 2.1 and 2.2, we first introduce the *J*-score and then we describe the adaptive search based on sequential halving.

### 2.1 *J*-score

We first introduce some notation. For a sequence *s*, let |*s*| denote the length of *s*. A *k*-mer of a string *s* is simply a length-*k* substring of *s*. For a given sequence *s*, let $N_{k}(s)$ be the set of *k*-mers in *s*. For a set *S* of sequences, let $N_{k}(S)=\cup_{s\in S}N_{k}(s)$. For a sequence *s* and *k*-mer *a*, let $c_{s}(a)$ be the number of times *a* appears in *s*. For a set of sequences *S*, let $c_{S}(a)=\frac{1}{\left|S\right|}\sum_{s\in S}c_{s}(a)$. Let *n* be the number of query sequences, *m* be the number of HMMs, and ℓ be the maximum length over all sequences in the dataset.

In the original UPP pipeline (Nguyen *et al.* 2015), each of the HMMs is built from a subset of the backbone sequences. For an HMM *h*, we let $S_{h}$ be the subset of backbone sequences used by UPP to create the HMM (which is done using HMMer; Finn *et al.* 2011). Our similarity score J(s,h) can be thought of as a kind of weighted Jaccard similarity (Jaccard 1912) between the *k*-mers in *q* and in $S_{h}$. We formally define it as

(1) $$J(q,h)=\frac{\sum_{a\in N_{k}(q)}\min(c_{q}(a),\;c_{S_{h}}(a))}{(|q|-k+1)+\frac{\sum_{s\in S_{h}}\left(|s|-k+1\right)}{\left|S_{h}\right|}}.$$

Each *k*-mer *a* that appears in both *q* and $S_{h}$, contributes an additive term of $\min(c_{q}(a),c_{S_{h}}(a))$ to the numerator in (1). This can be thought of as a kind of intersection between the *k*-mers of *q* and the *k*-mers of an “average” of the sequences in $S_{h}$ (since $c_{S_{h}}$ has a normalization factor of $\left|S_{h}\right|$). The denominator is simply the number of *k*-mers in *q* plus the average number of *k*-mers in $S_{h}$. The *J*-score is inspired by the *k*-mer Jaccard similarity and its usefulness in estimating pairwise sequence alignment scores (Berlin *et al.* 2015, Jain *et al.* 2018, Kamath *et al.* 2020). In particular, the *J*-score is equivalent to the multiset Jaccard similarity (Rajaraman and Ullman 2011), except that each *k*-mer can appear a rational number of times in a multiset.

**Figure 3.**
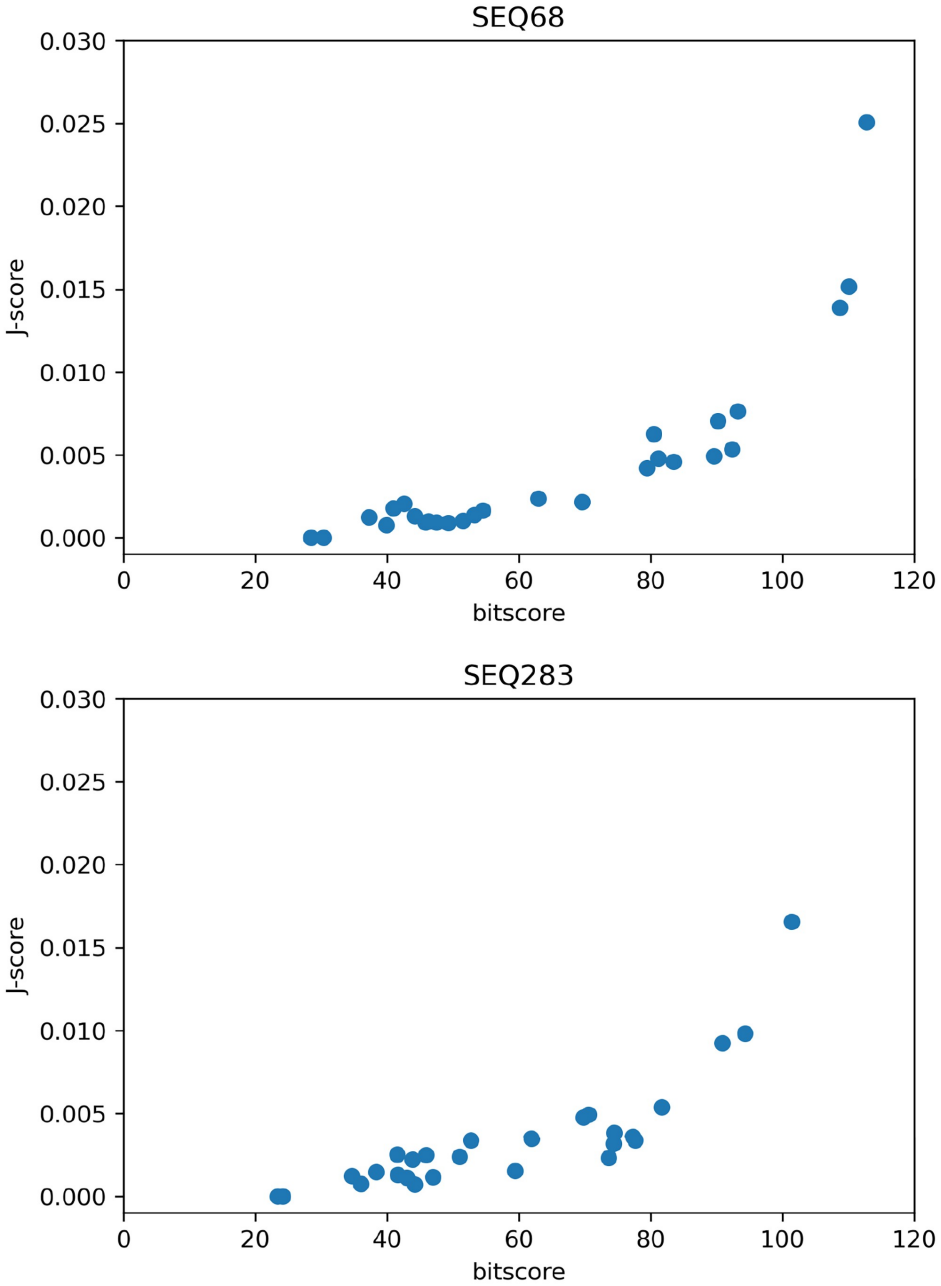
Scatterplot of our proposed *J*-score when k=9 versus the bit-score (the score which UPP attempts to maximize). Each point corresponds to the scores for sequences 68 and 283 from the AMINO test dataset (included with UPP; Nguyen *et al.* 2015) and one of the HMMs created with a backbone of size 100. Observe the correlation between bitscore and *J*-score.

We propose to perform the query-to-HMM assignment based on the *J*-score, i.e. assigning query *q* to

(2) $$h^{*}=\arg\underset{h}{\max}J(q,h),$$

instead of doing this assignment based on the bit-score (which corresponds to the probability of the HMM generating the query sequence), which is employed in UPP. As we verify empirically (see Fig. 3), the *J*-score is roughly monotonically increasing in the bit-score (which is the score UPP utilizes to perform the query-to-HMM assignment). This monotonic trend tends to hold particularly well for larger values of *J*-score/bit-score, which is what is important when trying to choose $\arg\;\max_{h}J(q,h)$.

We analyze the relationship between J-score and bitscore in detail for the 16S.3 and 16S.T nucleotide datasets from the Comparative Ribosomal Website (Cannone *et al.* 2002), and the adh amino acid dataset from homfam (Sievers *et al.* 2011). The 16S.3 dataset has 6323 sequences. Using a backbone size of 1000, UPP produces 271 HMMs. The UPP algorithm therefore compares 5323 sequences to 271 HMMs. The 16S.3 dataset has 7350 sequences, and the adh dataset contains 21 331 sequences. The average sequence lengths of 16S.3, 16S.T and adh are 1492, 1557, and 124, respectively. We define $d_{0}$ to be the fraction of query sequences where the top-scoring HMM according to bitscore is the top-scoring HMM according to J-score. We define $d_{x}$ to be the fraction of query sequences where the top scoring HMM according to bitscore is among the top *x* percent of HMMs according to J-score. For each query sequence, we also compute the ordering of the HMMs according to bitscore, and the ordering of the HMMs according to J-score. We then compute the Spearman’s rank correlation coefficient (Spearman 2015) between the bitscore HMM ordering and the J-score HMM ordering, along with its associated *P*-value. This correlation coefficient measures how well the relationship between the J-score and the bitscore can be described using a monotonic function. The Spearman coefficient ranges from −1 to 1, with 0 implying no correlation, and 1 implying an exact monotonic relationship. We report this information for the three datasets in Table 1. Observe that in Table 1, the value of *k* that causes the J-score to correlate best with bitscore is lower for the amino acid dataset adh compared to the two nucleotide datasets. This is because it is harder for the short amino acid query sequences in adh to share long *k*-mers with the backbone sequences used to form the HMMs, and thus yield non-zero J-scores. It is harder for adh query sequences to share long *k*-mers with the backbone sequences because there are 20 amino acids as opposed to four nucleotides and because the sequences in adh are much shorter and therefore have far less kmers than sequences in the 16S datasets. When a query sequence does not share any *k*-mers with any backbone sequences, the J-scores are zero for all HMMs, and thus do not correlate well with the bitscores (which are generally not constant across HMMs).

**Table 1.** Statistics showing how well the *J*-score correlates with bitscore on 16S.3, 16S.T, and adh datasets.^a^

Dataset	*k*	$d_{0}$	$d_{10}$	Spearman	*P*-value
16S.3	10	0.502	0.779	0.547	.002
	15	0.587	0.835	0.563	.004
	20	0.619	0.837	0.618	.001
16S.T	10	0.445	0.718	0.500	.007
	15	0.490	0.724	0.545	.005
	20	0.475	0.656	0.570	.116
adh	5	0.616	0.885	0.472	.001
	10	0.527	0.746	0.246	.271
	15	0.409	0.572	0.153	.511
	20	0.341	0.490	0.120	.607

a The backbone sizes used are 1000 for all datasets. The Spearman coefficients and corresponding *P*-values are averaged across all query sequences in a dataset. The Spearman coefficient and corresponding *P*-value is not defined for a query sequence when the J-scores for that query sequence are equal to some constant for all HMMs. Therefore, if a query sequence does not share any *k*-mers with any backbone sequences, the J-score is 0 for all HMMs, and the corresponding Spearman Coefficient is not defined. If the Spearman Coefficient is not defined, we set the Spearman Coefficient to 0, and set the *P*-value to 1 to be as adversarial as possible when computing the averages in the table.

We note that the *J*-score can be computed naively for all pairs of query sequences and HMMs in amortized O(nmℓ) time by building a hash table for *q* that maps each *k*-mer *a* present in *q* to $c_{s}(a)$, and building a hash table that maps each *k*-mer *a* present in $S_{h}$ to $c_{S_{h}}(a)$. The summation in the numerator in (1) can then be computed in amortized O(ℓ) time.

While the specific form of the score in (1) may seem arbitrary, our main motivation for working with it is that it allows for the computation of unbiased estimates of J(q,h) from randomly selected *k*-mers from $N_{k}(q)$. Let $P_{q}$ be a distribution that chooses each *k*-mer in the set $N_{k}(q)$ with equal probability (i.e., with probability $P_{q}(a)=|N_{k}(q){|}^{-1}$ for $a\in N_{k}(q)$). For a batch of *k*-mers $B=\{a_{1},\ldots ,a_{B}\}$ of size *B*, drawn i.i.d. according to $P_{q}$, one can build an estimator

(3) $$\tilde{J}(q,h,B)=\frac{\frac{\left|N_{k}(q)\right|}{B}\sum_{a\in B}\min(c_{q}(a),\;c_{S_{h}}(a))}{(|q|-k+1)+\frac{\sum_{s\in S_{h}}\left(|s|-k+1\right)}{\left|S_{h}\right|}}.$$

This is an unbiased estimator because

(4) $$\begin{array}{c} E\left[\tilde{J}(q,h,B)\right]=\frac{\frac{\left|N_{k}(q)\right|}{B}\sum_{t=1}^{B}E\left[\min(c_{q}(a_{t}),\;c_{S_{h}}(a_{t}))\right]}{(|q|-k+1)+\frac{\sum_{s\in S_{h}}\left(|s|-k+1\right)}{\left|S_{h}\right|}}\\ =\frac{\frac{\left|N_{k}(q)\right|}{B}\sum_{t=1}^{B}\sum_{a\in N_{k}(q)}P_{q}(a)\min(c_{q}(a),\;c_{S_{h}}(a))}{(|q|-k+1)+\frac{\sum_{s\in S_{h}}\left(|s|-k+1\right)}{\left|S_{h}\right|}}\\ =\frac{\sum_{a\in N_{k}(q)}\min(c_{q}(a),\;c_{S_{h}}(a))}{(|q|-k+1)+\frac{\sum_{s\in S_{h}}\left(|s|-k+1\right)}{\left|S_{h}\right|}}=J(q,h). \end{array}$$

Notice that, unlike for the *J*-score, the standard approach for estimating the Jaccard similarity $\frac{\left|A\cap B\right|}{\left|A\cup B\right|}$ between sets *A* and *B* is the use of min-hashes (Broder 1997, Broder *et al.* 2000, Berlin *et al.* 2015). What makes J(q,h) somewhat different from the Jaccard similarity is the fact that the denominator of J(q,h) does not require a set union calculation, it is just a function of sequence length. Thus, we only need to estimate the numerator of J(q,h) from samples.

### 2.2 Adaptive search via multi-armed bandits

Because we have an unbiased estimator for J(q,h) based on samples from $N_{k}(q)$, we can search for $h^{*}=\arg\;\max_{h}J(q,h)$ adaptively by iteratively sampling more *k*-mers from $N_{k}(q)$ in order to refine the estimate of J(q,h) for more promising HMM candidates *h*. Our goal is then to minimize the number of times we need to evaluate $\tilde{J}(q,h,\{a\})$ for a *k*-mer *a* in $N_{k}(q)$. We refer to the evaluation of $\tilde{J}(q,h,\{a\})$ as a “*k*-mer evaluation” on *h*.

The problem of finding $h^{*}=\arg\;\max_{h}J(q,h)$ while minimizing the total number of *k*-mer evaluations fits well within the MAB literature. In the MAB setting, there are several random variables (referred to as “arms”), and at each time step, we can sample one of the random variables (or “pull an arm”). In the best-arm identification problem (Jamieson and Nowak 2014), the goal is to identify the arm with the largest mean reward (with high probability) using as few arm pulls as possible. In our problem, each arm corresponds to an HMM, and pulling arm *h* corresponds to sampling a *k*-mer *a* from $N_{k}(q)$ uniformly at random, and evaluating $\tilde{J}(q,h,\{a\})$. This is a best-arm identification problem because we want to find $h^{*}=\arg\;\max_{h}J(q,h)$ by performing as few *k*-mer evaluations as possible.

Two well-known algorithms for accomplishing this are Upper-Confidence Bound (UCB) (Lai and Robbins 1985) and Sequential Halving (Karnin *et al.* 2013). The UCB algorithm is widely used in the literature and is amenable to a clean theoretical analysis of the number of arm pulls needed to identify the best arm with high probability (Lattimore and Szepesvári 2020). Sequential Halving (Karnin *et al.* 2013) is simpler to implement and achieves great results in many practical settings (Baharav and Tse 2019), although its theoretical analysis is less straightforward.

For this reason, we first state a theoretical result characterizing the number of *k*-mer evaluations needed to identify the best HMM $h^{*}=\arg\;\max_{h}J(q,h)$ when the UCB algorithm is applied to our problem, but use Sequential Halving in our software implementation due to its good performance in practice (Cazenave 2015, Pepels *et al.* 2016, Baharav and Tse 2019). We present the UCB-based algorithm as Algorithm 2 and its theoretical analysis in detail in Section 5. Under some regularity conditions (see Section 5), this analysis implies that the query-to-HMM assignment problem using *J*-scores can be solved very efficiently:

Corollary 1 *The optimal HMM* $h^{*}$in the search problem $h^{*}=\arg\;\max_{h}J(q,h)$*can correctly identified in time* O(mn log m)*with probability* 1−o(1).

While the UCB algorithm provides us with a time complexity that is independent of ℓ, for our practical implementation we utilize a simpler adaptive algorithm that still has a linear dependence on ℓ. The algorithm we implemented in the software is a modified Sequential Halving algorithm, and it is presented as Algorithm 1.

Algorithm 1 Adaptive search to find $h^{*}=\arg\;\max_{h}J(q,h)$

**Input:** *q*, $\left[S_{h}:h\in [1:m]\right]$, *B*, *R*, *T*

**Output:**$h^{*}$

1: $S_{\text{active}}\leftarrow \{1,\ldots ,m\}$

2: For all h∈[1:m], set ${\hat{J}}_{h}\leftarrow 0$

3: **for**r=1,…,R**do**

4:  Draw a batch of *k*-mers $B\subset N_{k}(q)$ of size *B* with replacement

5:  **for**$h\in S_{\text{active}}$**do**

6:   ${\hat{J}}_{h}\leftarrow \frac{1}{r}\left(\left(r-1){\hat{J}}_{h}+\tilde{J}(q,h,B\right)\right)$

7:  **end for**

8:  $t\leftarrow \max(|S_{\text{active}}|/2,T)$

9:  $S_{\text{active}}\leftarrow \{t\;\text{elements}\;\text{of}\;S_{\text{active}}\;\text{with}\;\text{highest}\;\text{values}\;\text{of}\;{\hat{J}}_{h}\}$

10: **end for**

11: Compute J(q,h) exactly for the *T* elements in $S_{\text{active}}$ with highest values of ${\hat{J}}_{h}$

12: **return**$h^{*}=\arg\min_{h\in S_{\text{active}}}J(q,h)$

Observe that Algorithm 1 takes O(mRB+Tℓ)*k*-mer evaluations and O(mRB+Tℓ) amortized time since we can pre-compute a hash-table mapping each *k*-mer $a\in N_{k}(S_{i})$ to $c_{S_{i}}(a)$ for each *i*, along with the analogous map for *q*. Applying this algorithm to all *n* query sequences requires O(nmRB+Tnℓ) amortized time. For *R*, *B*, *T* constant, the time complexity is O(nm+nℓ), which gives a better dependence on ℓ than UPP and UPP2. It also gives an improved run-time compared to a naive version of our algorithm that simply computes J(q,h) for all *q* and *h*, which requires O(nmℓ) time. In practice, we pick *R*, *B*, *T* depending on how confident we want to be in the selected $h^{*}$. Note that if a query sequence does not share a *k*-mer with any sequence in any of the HMMs, it is assigned to the HMM corresponding to all sequences in the backbone. We also point out that we parallelized the algorithm to make use of a user-specified number of cores. All of our code is written in Python, and is available at: https://github.com/ilanshom/adaptiveMSA. The additional scripts used to generate the results in this paper are also available at this link.

## 3 Results

### 3.1 Datasets and performance metrics

The first three nucleotide datasets we use are from the Comparitive Ribosomal Website (Cannone *et al.* 2002). They are named 16S.3, 16S.T and 16S.B.ALL. These three biological datasets were used in the UPP and UPP2 papers. The next three nucleotide datasets we test on were generated by Indelible (Fletcher and Yang 2009), and were introduced in (Mirarab *et al.* 2014). They are named 10000M2, 10000M3, and 10000M4 and were used in the UPP paper. The final three nucleotide datasets we test are called RNASim10000, RNASim50000, RNASim100000, and RNASim200000 and were introduced in Mirarab *et al.* (2014). These simulated datasets were tested in the UPP paper. The first three amino acid datasets we tested were generated using ROSE (Stoye *et al.* 1998) and introduced in Liu *et al.* (2009). The datasets are called ROSE1000S, ROSE1000M, and ROSE1000L. These datasets were used in both the UPP and UPP2 papers. Finally, we test 19 large HomFam amino acid datasets (Sievers *et al.* 2011). The 19 datasets in HomFam used are as follows: aat, Acetyltransf, adh, aldosered, biotin_lipoyl, blmb, ghf13, gluts, hla, hom, myb_DNA-binding, p450, PDZ, Rhodanese, rrm, rvp, sdr, tRNA-synt_2b, zf-CCHH. Each of these biological datasets has a reference alignment for a very small subset of the sequences (5–20 sequences, median 7). This is in contrast to all other datasets, which have full reference alignments. Information on the number of sequences and average sequence length for each dataset is present in Table 2. Note that both 16S and homfam include datasets with high levels of sequence length heterogeneity, which UPP is known to handle well (Nguyen *et al.* 2015). All datasets were obtained from this website: https://sites.google.com/eng.ucsd.edu/datasets/alignment/pastaupp.

**Table 2.** Results for all datasets.^a^

Dataset	Alg.	Time (s)	SP error	TC score	SP score	Modeler score
16S.B.ALL	UPP	20371	0.052	0.019	0.947	0.949
(27 643)	UPP2	9139	0.043	0.001	0.955	0.959
(1372)	J-bandit	1897	0.053	0.019	0.944	0.95
16S.T	UPP	7422	0.177	0.011	0.831	0.814
(7350)	UPP2	5179	0.197	0.005	0.792	0.815
(1492)	J-bandit	2759	0.198	0.009	0.789	0.816
16S.3	UPP	6710	0.122	0.006	0.924	0.832
(6323)	UPP2	4531	0.127	0.004	0.914	0.832
(1557)	J-bandit	2605	0.122	0.008	0.923	0.832
Indelible 10000M2	UPP	4718	0.075	0.02	0.908	0.941
(10 000)	UPP2	2988	0.06	0.016	0.927	0.952
(1000)	J-bandit	2477	0.062	0.007	0.924	0.952
Indelible 10000M3	UPP	3484	0.009	0.113	0.988	0.995
(10 000)	UPP2	1502	0.008	0.077	0.988	0.996
(1000)	J-bandit	3208	0.01	0.062	0.985	0.995
Indelible 10000M4	UPP	3853	0.003	0.395	0.996	0.998
(10 000)	UPP2	1470	0.004	0.411	0.995	0.998
(1000)	J-bandit	3342	0.007	0.115	0.99	0.996
RNASim 10000	UPP	11 015	0.096	0.003	0.903	0.906
(10 000)	UPP2	6681	0.097	0.004	0.902	0.905
(1555)	J-bandit	3213	0.106	0.003	0.887	0.901
RNASim 50000	UPP	48 182	0.099	0.002	0.9	0.903
(50 000)	UPP2	23 445	0.104	0.002	0.894	0.897
(1555)	J-bandit	3986	0.112	0.001	0.883	0.894
RNASim 100000	UPP	101 853	0.089	0.002	0.909	0.912
(100 000)	UPP2	43 334	0.09	0.003	0.908	0.911
(1554)	J-bandit	7168	0.109	0.002	0.884	0.899
ROSE 1000S1	UPP	1289	0.127	0.012	0.871	0.876
(1000)	UPP2	1022	0.191	0.001	0.807	0.812
(1025)	J-bandit	1061	0.171	0.0	0.825	0.833
ROSE 1000M1	UPP	1497	0.19	0.037	0.807	0.814
(1000)	UPP2	1383	0.455	0.01	0.539	0.552
(1058)	J-bandit	1405	0.211	0.009	0.784	0.793
ROSE 1000L1	UPP	1354	0.163	0.074	0.832	0.842
(1000)	UPP2	1681	0.323	0.027	0.669	0.685
(1079)	J-bandit	1145	0.188	0.024	0.809	0.816
homfam (19)	UPP	356	0.241	0.46	0.873	0.78
(27 091)	UPP2	197	0.246	0.47	0.923	0.768
(144)	J-bandit	272	0.254	0.438	0.876	0.781

a For all datasets, c=0.2,R=3,T=10 for the J-bandit runs. The number of sequences is in parentheses below dataset name, followed by average sequence length in parentheses [for homfam (19), these statistics are averaged over the 19 datasets]. The times reported for homfam (19) do not include backbone generation.

We report SP-error, SP-score, modeler-score, TC-score. In an MSA A, consider sequences $q_{1}$ and $q_{2}$. The *i*th symbol in $q_{1}$ is said to be homologous to the *j*th symbol in $q_{2}$ if they appear in the same column in A. In this case, $q_{1}[i]$ and $q_{2}[j]$ are said to form a homologous pair. For a reference alignment A and an estimated alignment $A^{\prime },$ the SPFN rate is the fraction of homologous pairs in A that are not present in $A^{\prime }.$ The SP-score is defined as 1 - SPFN, and is a measure of recall. The SPFP rate is the fraction of homologous pairs in $A^{\prime }$ that are not present in A. The Modeler score is defined as 1 − SPFP and is a measure of precision. The SP error is equal to the average of the SPFN rate and the SPFP rate (Nguyen *et al.* 2015). We define the Total Column score (TC-score) as the number of columns in $A^{\prime }$ that are present in A, divided by the total number of columns in A. We use FastSP (Mirarab and Warnow 2011) to calculate all metrics.

### 3.2 Experiments

We test our algorithm, UPP, and UPP2 on all datasets mentioned above. UPP and UPP2 are already compared extensively with existing MSA packages, so we focus on comparing our algorithm with UPP and UPP2. Throughout this section, we refer to our modified version of UPP that makes use of Algorithm 1 to estimate the best HMM for each query sequence as J-bandit. We refer to the modified version of UPP that assigns each query sequence to the HMM with the highest J-score as J-exact. We run all algorithms by generating an alignment of the backbone sequences using PASTA (Mirarab *et al.* 2014, 2015). Using PASTA for this task is the only option included in UPP and UPP2. The backbone is selected randomly from the set of input sequences whose length is within 25% of the length of the median sequence length as is standard in UPP. For all algorithms in all experiments, we specified that 24 processors can be used. We ran all simulations on a machine with 80 physical cores, 160 threads, and 512 GB of memory. We report SP error, SP score, modeler score, TC score, and time taken by the algorithm from start to finish (including backbone generation). We also report peak memory usage for a subset of the datasets.

**Figure 4.**
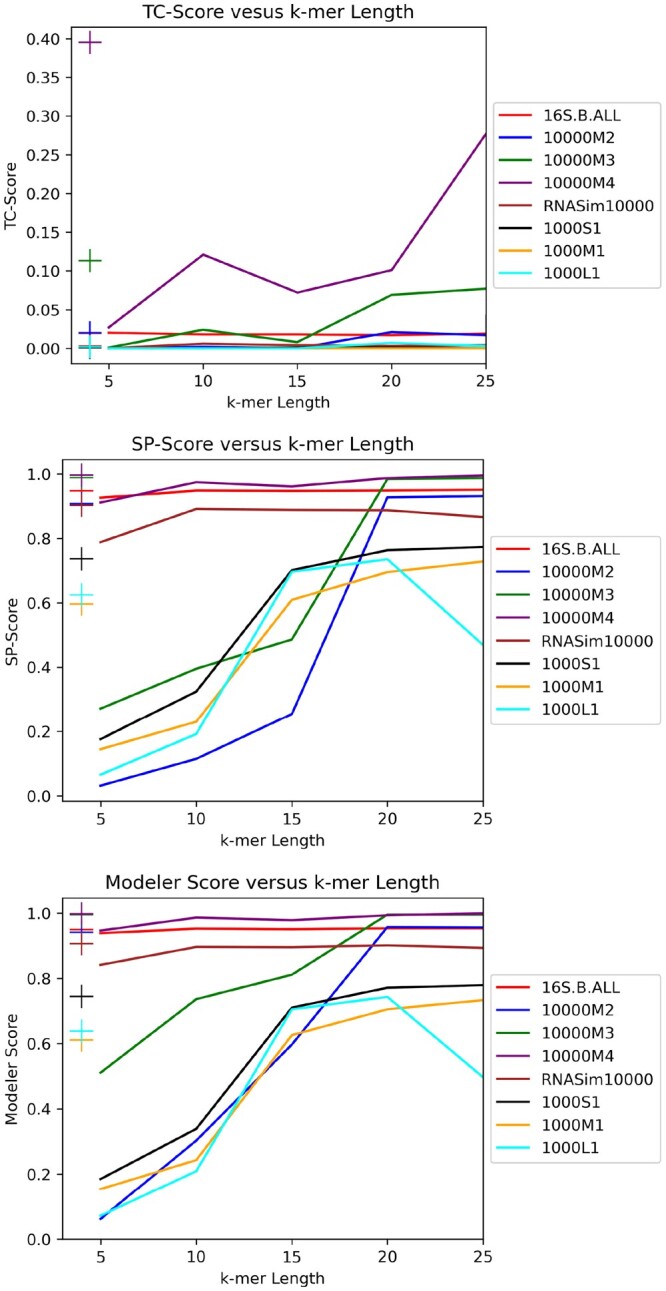
Performance metrics for various datasets when the J-score is computed exactly for a range of *k*. The “+” symbols correspond to the performance metric for UPP for the dataset corresponding to the symbol’s color.

We began by running J-exact for *k* values of 5, 10, 15, 20, and 25 on 16S.B.ALL, Indelible 10000M2, Indelible 10000M3, Indelible 10000M4, RNASim10000, ROSE 1000S1, ROSE 1000M1, and ROSE 1000L1. These datasets were chosen to include a mix of nucleotide and amino acid datasets. We did not include any homfam datasets in this experiment because they only have reference alignments for very small subsets of the sequences, and because the sequences are very short in comparison to the other datasets (the average of the average sequence lengths of the homfam datasets was 144, while the average sequence length of all other datasets was at least 1000). We made sure to include 16S.B.ALL in the experiment because it is a biological dataset as opposed to simulated, and because it displays substantial sequence length heterogeneity, which UPP is known to handle well (Nguyen *et al.* 2015). For the 16S, Indelible, and RNASim datasets, we used a backbone of 1000, while for ROSE datasets, we used a backbone of 100. We observed that for all performance metrics, setting *k* to 20 gave comparable performance to UPP, as shown in Fig. 4. We therefore set *k* to 20 in proceeding experiments, with the exception of the homfam datasets which have sequences of much shorter length than the other datasets.

Next, we ran J-bandit on 16S.B.ALL for a range of parameters in Algorithm 1 to observe their effect on performance and runtime. We chose 16S.B.ALL because it is one of the largest datasets we had in terms of the number of sequences and the sequence lengths, it has substantial sequence length heterogeneity, and because it is biological (as opposed to simulated). For the sequence *q*, the batch size *B* is chosen to be c·|q| where |*q*| is the length of *q* and *c* is a constant. We tested *c* values of 0.1, 0.2, and 0.3, and tested *R* values of 2, 3, and 4. We kept *k* fixed at 20, and *T* fixed at 10 for these experiments. The performance on 16S.B.ALL does not change much for the various values of *c* and *R* that we tested and remains close to the performance of UPP and J-Exact. The runtime does not seem to change significantly either across the parameter settings but is significantly lower than UPP and J-Exact. Based on these observations, we choose *c* to be 0.2, *R* to be 3, and T to be 10 for J-bandit in all of the proceeding runs of J-bandit.

Finally, we ran J-bandit on a wide range of datasets and compared its performance to UPP and UPP2. All datasets in Section 3.1 other than RNASim 200000 were tested and the SP error and all performance metrics along with time taken were calculated and are shown in Table 2. We did not compare the algorithms on RNASim 200000 because due to the fact that such a comparison would use excessive computing time: UPP used over 28 h to run on RNASim 100000 and the runtime of all three algorithms scales roughly linearly with the number of query sequences. For all datasets, J-bandit used parameters c=0.2, R=3, and T=10. For all datasets besides those in homfam (19), we set *K* to 20. For the 19 large homfam datasets, we set *K* to 10 because these sequences have a much shorter average length of 144, with many sequences shorter than 20 (the average sequence length of all other datasets was at least 1000). Note, however, that setting *K* to 20 for the 19 homfam datasets did not change the overall average performance by much. We used a backbone size of 1,000 for all datasets except for the three ROSE datasets since these datasets only have 1000 sequences. For these three ROSE datasets, we used a backbone size of 500 sequences.

To summarize the results in Table 2, J-bandit runs faster than UPP, creating alignments with similar, though often slightly degraded accuracy. Compared to UPP2, J-bandit sometimes gives improved accuracy and sometimes gives degraded accuracy depending on the dataset. Similarly, J-bandit is faster than UPP2 in most, but not all cases.

**Figure 5.**
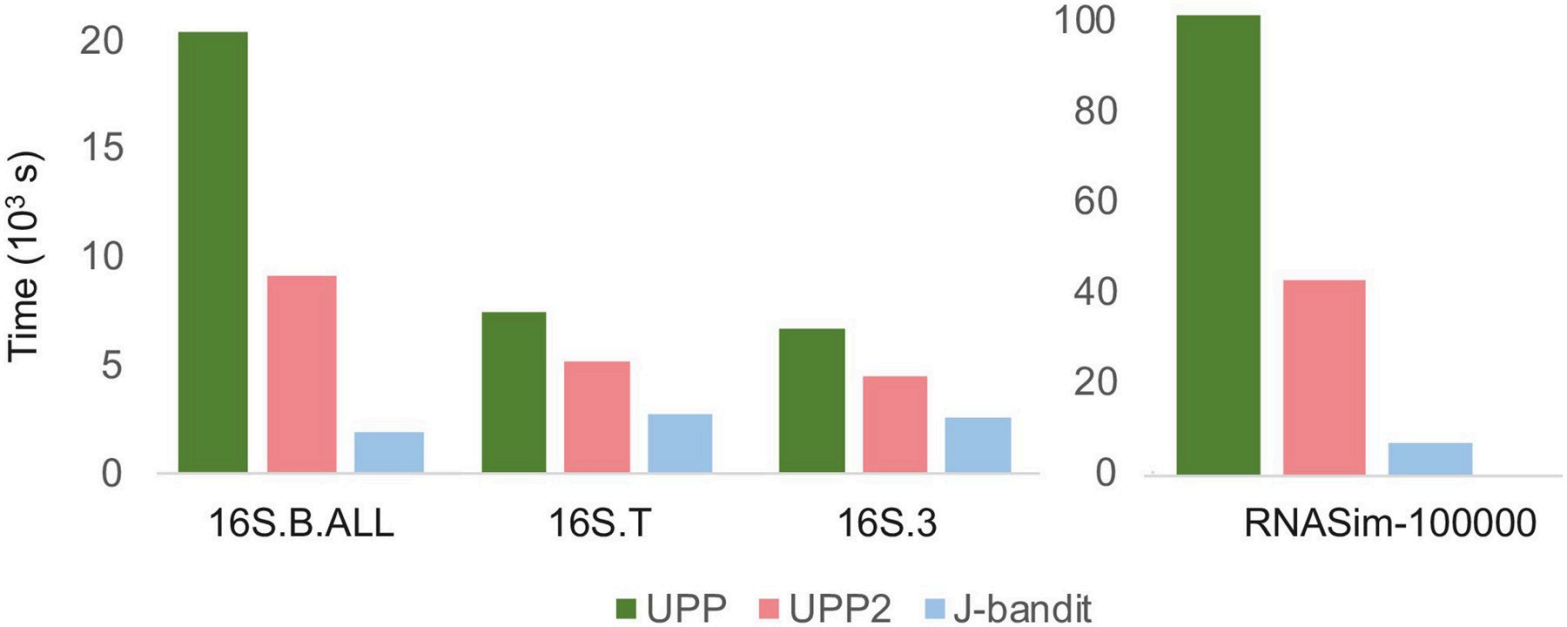
Time (in seconds) taken for several datasets with long sequence lengths.

For datasets with large sequence lengths and many sequences, e.g. 16S datasets and RNASim datasets, J-bandit is significantly faster than both UPP and UPP2 as highlighted in Fig. 5. For example, for the RNASim-100000 dataset, J-bandit completes in 16.5% of the time used by UPP2, and only 7% of the time used by UPP. This is expected from a theoretical standpoint since both UPP and UPP2 calculate bitscore, which requires quadratic time in the sequence length ℓ, whereas even exact computation of the J-score requires only linear amortized time in ℓ. For datasets with shorter sequence lengths such as the three Indelible datasets, the three ROSE datasets, and the 19 homfam datasets, we observe the increase in speed of J-bandit is not as pronounced compared to UPP and UPP2. In some cases, J-bandit is even slower than UPP2 (e.g. Indelible 10000M3, 10000M4). This is not surprising, since UPP2 is designed to be faster than UPP, though unlike J-bandit, it is not designed to reduce the effect of sequence length on runtime. It should be noted that our algorithms are written in Python, while HMMer (Finn *et al.* 2011), which is used for making the assignment in UPP and UPP2, is written in C. Hence, it may be possible to accelerate our *J*-score-based approach even further by writing it in a low-level programming language like C.

We also performed a smaller experiment to assess peak memory usage on four large datasets. In Table 3, we show the peak memory usage of UPP, UPP2, and J-bandit on 16S.3, Indelible 10000M2, RNASim 10000, and ROSE 1000M1. We chose these four large datasets because we are most interested in memory usage when a lot of data need to be stored, in both the nucleotide and amino acid cases. We obtained these results using the “memory-profiler” Python package. The paramater settings used in these runs are identical to those used in the runs presented in Table 2. We observe that J-bandit has a higher peak memory usage than UPP for all datasets. In comparison to UPP2, J-bandit has a higher memory usage on some datasets and a lower memory usage on other datasets. UPP uses less memory than J-bandit due to the fact that J-bandit creates hash tables that store all *k*-mers in the query sequences and backbone sequences in order to efficiently estimate the J-score. In contrast, these hash tables are not created for UPP and UPP2. In addition, the memory-intensive computation of estimating and computing the J-score is implemented in Python in J-bandit, whereas bitscore computation in UPP and UPP2 is performed by HMMer which is written in C, a language that generally uses less memory than Python.

**Table 3.** Peak memory usage results in megabytes (MB) for four datasets.^a^

Dataset	Alg.	Peak memory (MB)
16S.3	UPP	5103
(6323)	UPP2	24 098
(1557)	J-bandit	9892
Indelible 10000M2	UPP	4265
(10 000)	UPP2	13 851
(1000)	J-bandit	28 102
RNASim	UPP	7076
(10 000)	UPP2	24 445
(1555)	J-bandit	26 594
ROSE 1000M1	UPP	2077
(1000)	UPP2	2020
(1058)	J-bandit	8611

a For all datasets, c=0.2,R=3,T=10 for the J-bandit runs. The number of sequences is in parentheses below dataset name, followed by average sequence length in parentheses.

## 4 Conclusion

In this work, we proposed a method to speed up the query-to-HMM assignment step in the UPP pipeline. This strategy is based on two key ingredients: the introduction of the *J*-score and an adaptive search algorithm inspired by Multi-Armed Bandit algorithms. This allows us to achieve theoretical and practical reductions in run-time when replacing the query-to-HMM module in UPP with our proposed approach.

While the techniques introduced were developed for the specific setting of the UPP pipeline, we believe that they may be of broader interest in bioinformatics since bitscores are used to choose the best HMM in many applications including orthology detection, and metagenomic pipelines. The *J*-score can be thought of as a kind of Jaccard similarity between a sequence and a set of sequences and can be easily generalized to measure similarity between two sets with different numbers of sequences. As we verified empirically, this score can be used as a proxy for the bit-score between a sequence and an HMM, in situations where exact calculation of the bit-score may not be needed. Finally, we point out that techniques from MAB may be applicable to other MSA pipelines.

## 5 Theoretical guarantees via the batched UCB algorithm

In this section, we describe how a version of the UCB algorithm (Lai and Robbins 1985) can be used to show that each query *q* can be assigned to the best HMM based on *J*-score in time O(m log m). In particular, we will use a batched version of the UCB algorithm (see, e.g. Tiwari *et al.* 2020), which is appropriate for the *J*-score refinement based on *k*-mer batches.

The batched UCB algorithm adapted to our problem is given by Algorithm 2. Similar to the standard UCB algorithm (Lai and Robbins 1985), the algorithm assumes that for each HMM *h* and a random *k*-mer *a*, the random variable $\tilde{J}(q,h,\{a\})$ is σ-sub-Gaussian, and that the parameter σ (or an upper bound) is known. Recall that a random variable *X* is σ-sub-Gaussian if $\mathrm{Pr}(X\;>\;t)\;\leq \;2\;\text{exp}(-t^{2}/\sigma^{2})$. Observe that $\tilde{J}(q,h,B)$ is trivially sub-Gaussian because it takes values in a finite set. In this case, an upper bound on the random variable $\tilde{J}(q,h,B)$ has a subgaussianity parameter $\frac{1}{2}(\max_{a\in N_{k}(q)}\tilde{J}(q,h,\{a\})-\min_{a\in N_{k}(q)}\tilde{J}(q,h,\{a\}))$. An upper bound on this quantity that can be used in place of it in the algorithm is $\frac{1}{2}\max_{a\in N_{k}(S_{i})}c_{S_{i}}(a)$ and can be found in a preprocessing step on the sets.

Algorithm 2 Batched UCB algorithm to find $h^{*}=\arg\;\max_{h}J(q,h)$

**Input:** *q*, $\left[S_{i}:i\in [1:m]\right]$, σ

**Output:**$h^{*}$

1: $S_{\text{active}}\leftarrow \{1,\ldots ,m\}$,   $t_{\text{used}}\leftarrow 0$,   C←∞

2: For all h∈[1:m], set ${\hat{J}}_{h}\leftarrow 0$

3: **while**$t_{\text{used}}<m$ and $|S_{\text{active}}|>1$**do**

4:  Draw a batch of *k*-mers $B\subset N_{k}(q)$ of size *B* with replacement

5:  **for**$h\in S_{\text{active}}$**do**

6:   ${\hat{J}}_{h}\leftarrow (t_{\text{used}}{\hat{J}}_{h}+\tilde{J}(q,h,B))/(t_{\text{used}}+B)$

7:   $C\leftarrow \sigma \sqrt{\frac{2\;\text{log}(1/\delta )}{t_{\text{used}}+B}}$

8:  **end for**

9:  $S_{\text{active}}\leftarrow \{h:{\hat{J}}_{h}+C\;\geq \;\max_{y}{\hat{J}}_{y}-C\}$

10:  $t_{\text{used}}\leftarrow t_{\text{used}}+B$

11: **end while**

12: **if**$|S_{\text{active}}|=1$**then**

13:  **return**$h^{*}\in S_{\text{active}}$

14: **end if**

15: Compute J(q,h) exactly for all $h\in S_{\text{active}}$

16: **return**$h^{*}=\arg\min_{h\in S_{\text{active}}}J(q,h)$

Algorithm 2 works by maintaining a set $S_{\text{active}}$ of active arms (HMMs), initialized as {1,…,m}. For each HMM $h\in S_{\text{active}}$, an estimate ${\hat{J}}_{h}$ of J(q,h) is maintained. At each iteration, a random *k*-mer batch of size *B* is drawn (with replacement) from $N_{k}(q)$ and the estimates ${\hat{J}}_{h}$ is updated for all $h\in S_{\text{active}}$. At the end of each iteration, we eliminate all *h* whose confidence interval does not intersect with the confidence interval of the current best candidate $\max_{y}{\hat{J}}_{h}$.

Once one HMM is left in $S_{\text{active}}$ (or $t_{\text{used}}\geq m$), we output it. Notice that this algorithm is similar to Algorithm 1, except that a more careful elimination criterion is used at the end of each round, based on confidence intervals. This allows us to obtain a theoretical guarantee for Algorithm 2. For h∈[1:m], let $\Delta_{h}=J(q,h^{*})-J(q,h)$. Then we have

Theorem 1 *For* $\delta =m^{-3}$*, with probability at least* $1-\frac{2}{\delta }$*, the algorithm returns the best HMM* $h^{*}=\arg\;\max_{h}J(q,h)$*using a total of M k-mer evaluations, where*

(5) $$M\;\leq \;\sum_{h=1}^{m}\min\left(\frac{24\sigma^{2}}{\Delta_{h}^{2}}\text{log}\;m\;+\;B,m\;+\;\ell \right).$$

Notice that if $\sigma /\Delta_{h}$ is Θ(1), then the algorithm finds $h^{*}$ with O(m log m)*k*-mer evaluations. Since we can precompute a hash-table mapping each *k*-mer $a\in N_{k}(S_{i})$ to $c_{S_{i}}(a)$ along with an analogous map for *q*, we can find $h^{*}$ with high probability in O(m log m) amortized time. Finding $h^{*}$ for all query sequences *q* results in an amortized run-time of O(nm log m), as we state in Corollary 1 in Section 2. This removes the dependence on ℓ completely (while UPP and UPP2 have a quadratic dependence on ℓ) and also improves upon the naive exhaustive search algorithm that computes each J(q,h) exactly in time O(nmℓ).

**Proof.** Notice that $t_{\text{used}}$ keeps track of how many *k*-mers have been used in the estimates ${\hat{J}}_{h}$, for $h\in S_{\text{active}}$. Since $\tilde{J}(q,h,\{a\})$ is σ-sub-Gaussian, Hoeffding’s inequality implies that, at any iteration of the algorithm and for any *h*,

(6) $$\mathrm{Pr}(|J(q,h)\;-\;{\hat{J}}_{h}|\;>\;C)\;\leq \;2\;\text{exp}\;\left(-\frac{t_{\text{used}}C^{2}}{2\sigma^{2}}\right)=2\delta ,$$

where the equality follows since $C=\sigma \sqrt{\frac{2\;\text{log}(1/\delta )}{t_{\text{used}}}}$. Due to the constraint $t_{\text{used}}<m$ in the while loop, at most m/B iterations occur, and at most $m(m/B)\leq m^{2}$ estimates ${\hat{J}}_{h}$ are computed throughout the whole algorithm. Hence, from the union bound we have that (6) holds for all estimates with probability at most $m^{2}(2\delta )$. By setting $\delta =1/m^{3}$, we have that $J(q,h)\in [{\hat{J}}_{h}-C,{\hat{J}}_{h}+C]$ for all $h\in S_{\text{active}}$ in all iterations of the algorithm, with probability at least $1-m^{2}(2\delta )=1-\frac{2}{m}$. The fact that $J(q,h)\in [{\hat{J}}_{h}-C,{\hat{J}}_{h}+C]$ for all $h\in S_{\text{active}}$ implies that $h^{*}$ can never be eliminated and must be in $S_{\text{active}}$ at the end of the algorithm.

Now consider some $h\neq h^{*}$. Suppose $t_{\text{used}}>\frac{6}{\Delta_{h}^{2}}{\left(2\sigma \right)}^{2}\;\text{log}\;m=\frac{2}{\Delta_{h}^{2}}{\left(2\sigma \right)}^{2}\;\text{log}(m^{3})$. Then

(7) $$\Delta_{h}>(2\sigma )\sqrt{2\;\text{log}(m^{3})/t_{\text{used}}}=2\sigma \sqrt{2\;\text{log}(1/\delta )/t_{\text{used}}}=2C.$$

Since $\Delta_{h}=J(q,h^{*})-J(q,h)$, this implies that

(8) $$J(q,h^{*})\;-\;C\;>\;J(q,h)\;+\;C,$$

which guarantees that *h* is eliminated from $S_{\text{active}}$ if $t_{\text{used}}>\frac{6}{\Delta_{h}^{2}}{\left(2\sigma \right)}^{2}\;\text{log}\;m$.

If after m/B iterations, *h* is not eliminated, we must have $|S_{\text{active}}|\;>\;1$, and we will use an additional ℓ*k*-mer evaluations to compute J(q,h) exactly (on top of the B·(m/M)=m performed so far). Therefore, the number of *k*-mer evaluations $M_{h}$ required to remove *h* from $S_{\text{active}}$ satisfies

(9) $$M_{h}\;\leq \;\min\left[\frac{6}{\Delta_{h}^{2}}{\left(2\sigma \right)}^{2}\;\text{log}(m)+B,m+\ell \right]$$

for all *h* with probability 1−2/m. This yields the total number of *k*-mer evaluations in Theorem 1. □

## Supplementary Material

btae225_Supplementary_Data
