# Supplementary material for: Fast multiple sequence alignment via multi-armed bandits
Source: Bioinformatics. 2024 Jun 28;40(Suppl 1):i328–36. doi: 10.1093/bioinformatics/btae225 (PMC11211838; doi:10.1093/bioinformatics/btae225)
Supplement: btae225_Supplementary_Data [file btae225_supplementary_data.zip › btae225_Supplementary_Data/Mazooji.316.sup.1.pdf.pdf]

Fast Multiple Sequence Alignment via Multi-Armed Bandits: Supplement

Kayvon Mazooji and Ilan Shomorony

University of Illinois at Urbana-Champaign, Urbana, IL, USA
{mazooji2,ilans}@illinois.edu

1 Additional details on code and data

We used the following commands to run UPP and UPP2:

– UPP

```
run_upp.py -A 10 -B 1000 -M -1 --molecule amino -s <data_file> -x 24
```

– UPP2

```
run_upp.py -A 10 -B 1000 -M -1 --molecule amino -s <data_file>
   --decomp_only True --bitscore_adjust True --hier_upp True --early_stop True -x 24
```

All datasets were obtained from this website:

– https://sites.google.com/eng.ucsd.edu/datasets/alignment/pastaupp

Our code is available at:

– https://github.com/ilanshom/adaptiveMSA

2 Additional Figures

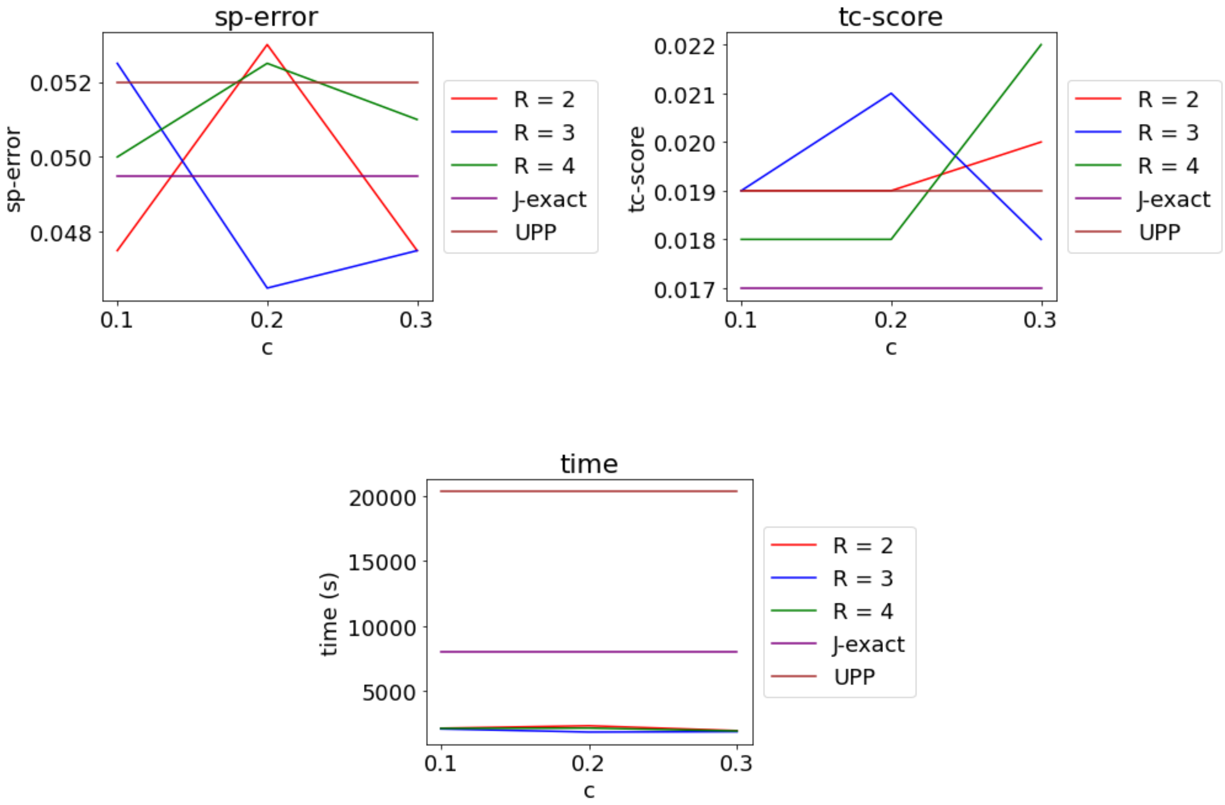

Fig. 1: Performance of J-bandit on 16S.B.ALL for a range of c where $B = c \cdot |q|$ for a sequence q, for different value of R. K was fixed to be 20 and T was fixed to be 10.
