# Supplementary material for: Fast multiple sequence alignment via multi-armed bandits
Source: Bioinformatics. 2024 Jun 28;40(Suppl 1):i328–36. doi: 10.1093/bioinformatics/btae225 (PMC11211838; doi:10.1093/bioinformatics/btae225)
Supplement: btae225_Supplementary_Data [file Mazooji.316.cover.pdf.pdf › btae225_Supplementary_Data/Mazooji.316.cover.pdf.pdf]

We thank all reviewers for their time and insightful comments. We made the following changes to the manuscript specified in the Metareview:

- We added an experiment to asses peak memory usage for J-bandit, UPP, and UPP2 in the Experiments section.
- We improved all the plots to make them more clear according to the suggestions of Reviewer 4.
- We added a more detailed discussion on the results in Table 2. In particular, we point out the small reduction in accuracy of J-bandit when compared to UPP, and the fact that J-bandit is faster than UPP and UPP2 when the sequence lengths are above a certain point, but isn't necessarily faster than UPP2 when the sequences are shorter.
- For each experiment, we added discussion on why the datasets used were chosen in the Experiments section.

Also, because we had extra space in the manuscript once it was put in OUP format, we moved the theoretical results from the supplement to a new section in the manuscript. Below we provide a point-by-point response to each of the reviews.

1 Reviewer 1

1.1. **Some typos: Page 4, line -12: measures how measures how Line -3 before Corollary 0.1: in the supplement Under some Corollary 0.1: can *be* correctly identified**

Thank you for pointing these typos out, we fixed them in the updated manuscript.

2 Reviewer 2

2.1. **The abstract claims a significant reduction in computation time as compared to UPP. However, according to Table 2 this is not true in all datasets.**

In the Experiments section, when discussing the results in Table 2, we added discussion on this point, and emphasize that it is for datasets with long sequences (e.g. 16S.B.ALL and RNASim10000) where we see significant speedup compared to UPP. We also mention this point about long sequences in the abstract and the introduction.

2.2. **It would be nice to see also the peak memory usage of the methods.**

At the end of the Experiments section, we added a table showing the peak memory usage of J-bandit, UPP, and UPP2 on several datasets with a large number of long sequences. We add some discussion on why the peak memory usage appears to be higher for J-bandit compared to UPP. On the other hand, there doesn't appear to be a clear trend in the comparison between the peak memory usage of J-bandit and that of UPP2.

3 Reviewer 3

3.1. **Table II is quite detailed, but tells a more nuanced story than does any of the main-paper text suggests. Why does J-bandit sometimes take longer than the other less heuristic approaches? Is there a concrete justification for the statement that "on average J-bandit delivers very similar performance to UPP and UPP2 in general"? It seems to me more accurate to say that J-bandit offers a typical heuristic trade: greater speed for lower accuracy compared to UPP & UPP2.**

We added a more detailed discussion on the results in Table 2 in the Experiments section addressing all of these points. While there is no dataset in Table 2 where J-bandit is slower than UPP, we do observe that J-bandit is noticeably slower than UPP2 for two of the Indelible datasets and the homfam datasets (on average). This is not surprising, because UPP2 uses a heuristic to achieve greater speed than UPP.

However, the heuristic in UPP2 is not optimized for the regime where the sequences are very long, since it still computes bitscore for query sequences and HMMs which runs in quadratic time in the sequence length. We see that for all datasets with average sequence length that is significantly longer than that of the Indelible and homfam datasets, J-bandit is faster than UPP2. Additionally, J-bandit is slowed down by the fact that its sequence to HMM assignment step is written in Python, unlike HMMer which is used by UPP and UPP2, and is written in C.

We updated the discussion in the Experiments section to mention that while the accuracy of J-bandit is similar to that of UPP, there is usually a slight degradation in the accuracy. We mention that when comparing the accuracy of J-bandit with that of UPP2, there is not a clear trend one way or the other - for some datasets J-bandit is more accurate, and for some datasets UPP2 is more accurate.

3.2. **I see the authors' point that UPP and UPP2 are compared elsewhere to other MSA approaches. Still, the appeal of this paper is limited by the fact that no link is made beyond the UPP/UPP2/J-bandit approaches to the wider world of more widely used approaches like Clustal, MAFFT, etc.**

Thank you for this comment. Unfortunately, due to time limitations in the two week revision period and our need to focus on the required revisions, we were not able to add these comparisons.

3.3. **Section IIIA introduces various datasets (CRW, Indelible, RNASim etc.), but subsequently only certain subsets are used, without much explanation why. E.g. RNASim20000 was seemingly not included in any results, though it was introduced.**

When presenting each experiment in the Experiments section, we added discussion on why we used specific datasets. We believe this helps justify our results.

3.4. **Results on the correlation between J-score and bitscore were limited (Table I, Figure 3). For a more persuasive argument, this could have been measured across a more datasets, using the more typical k=20 setting.**

We have updated the manuscript to include results on the 16S.3 and 16S.T datasets (nucleotide), and the homfam adh dataset (amino acid). We tested several k values for all datasets (including $k = 20$) We again observed that for many query sequences, the top J-score was the top bitscore.

3.5. **It was mildly distressing to see fixed, universal choices for (k,c) across such a variety of datasets, including both simulated and real data that should exhibit e.g. diverse gap lengths.**

We wanted find a single choice of (k, c) that works well for a wide variety of datasets so that the user does not need to have much insight on how to select these parameters. We did experiment with methods that select k based on the dataset, and methods that select k for each query sequence independently. However, we were not able to find a method that delivers improved performance across all datasets in general. We believe that the development of such methods are good directions for future work.

4 Reviewer 4

3.1. **The authors mention the following: "While UPP gives improved alignment accuracy on large datasets, it is often slower than other widely used software packages such as MUSCLE [6], MAFFT [7], and Clustal-Omega [8]" but make no attempt at comparing their method with the mentioned available tools. It would be interesting to know how their new method compares against tools faster than UPP and UPP2 now that it surpasses these two.**

Thank you for this comment. Unfortunately, due to time limitations in this two week revision period and our need to focus on the required revisions, we were not able to add these comparisons. In our initial draft of the paper. We didn't initially compare our algorithm to Muscle, MAFFT, and Clustal Omega

because in the UPP paper, the accuracy of these methods was shown to be considerably worse in general than the accuracy of UPP on the datasets analyzed in our paper.

3.2. **- The figures in this paper could use several improvements: – In figure 3, the subplot on the right does not indicate the y-axis value for the highest point at the top right. – In figure 3, the subplot on the left has different x-axis tick values compared to the subplot on the right although they both range from 0 to 100, and the x values for the points on the left subplot are unclear. – Figure 4 should have improved titles such as "TC-Score versus k-mer Length." Alternatively, figure titles are removed altogether and explained in the caption. The labels for the x- and y-axes should be capitalized and the x-axis written as "K-mer Length" since it is unclear whether "k" refers to sequence length or a specific parameter in the method. – Figure 5 should indicate that the numbers on the y-axis represent time in seconds: "Time (s)." – All figures in the paper could be bigger for clarity.**

Thank you for the suggestions. We changed all figures to take these comments into account. We believe that these comments improved the clarity of the presentation of this data.

3.3. **Minor typo on page 7: "Informaton." Minor typo on page 8: "compared it's performance."**

Thank you for pointing these typos out, we fixed them in the updated manuscript.
